# SARS-CoV-2 Infection and Vaccination Coverage among Fragile Populations in a Local Health Area of Northern Italy

**DOI:** 10.3390/life12071009

**Published:** 2022-07-07

**Authors:** Giovanni Maifredi, Ilaria Izzo, Cinzia Gasparotti, Claudio Vito Sileo, HIV-CoV Group, Francesco Castelli, Eugenia Quiros-Roldan

**Affiliations:** 1 ATS Brescia (Brescia Health Protection Agency), 25124 Brescia, Italy; giovanni.maifredi@ats-brescia.it (G.M.); cinzia.gasparotti@ats-brescia.it (C.G.); claudio.sileo@ats-brescia.it (C.V.S.); 2 Department of Infectious and Tropical Diseases, University of Brescia and ASST Spedali Civili General Hospital, 25123 Brescia, Italy; izzo.ilaria@hotmail.it (I.I.); francesco.castelli@unibs.it (F.C.)

**Keywords:** COVID-19, vaccination, PLWH, migrants

## Abstract

Italy was dramatically hit by the COVID-19 pandemic, and the province of Brescia was one of the epicenters of the outbreak. Furthermore, Brescia has one of the highest incidences of people living with HIV (PLWH) and a substantial presence of migrants. We conducted a retrospective cohort study involving all citizens connected to the Brescia Health Protection Agency, assessing the SARS-CoV-2 burden, COVID-19 prevalence, and vaccination coverage. A total of 1,004,210 persons were included, 3817 PLWH and 134,492 foreigners. SARS-CoV-2 infection, hospitalizations and death were more frequent among Italians than foreigners. SARS-CoV-2 infections and deaths were more frequent in HIV-uninfected people than in PLWH. PLWH and foreigners were less likely to have a SARS-CoV-2 diagnosis compared to HIV-negative patients. Migrants were more likely to be hospitalized but had a lower risk of death compared to HIV-negative patients. Regarding vaccination, 89.1% of the population received at least one dose of vaccine, while 70.4% of the Italian citizens and 36.3% of the foreigner subjects received three doses of vaccine. Foreigners showed a lower risk of being diagnosed with SARS-CoV-2 but a higher risk of complications. HIV infection was not associated with a higher risk of SARS-CoV-2 severe manifestations compared to the general population. COVID-19 vaccine hesitancy was not different between PLWH and HIV uninfected people, but foreigners were more hesitant.

## 1. Introduction

Italy was one of the European countries more dramatically hit by the COVID-19 pandemic, with 13.9 million cases and 155,900 deaths as of 25 March 2022 [1]. Lombardy, the most populated and industrialized Italian region, was the epicenter of the outbreak at the beginning of the pandemic, and the provinces of Brescia and Bergamo accounted for most cases. The disease ranges from asymptomatic cases to acute respiratory distress syndrome and death. Comorbidities and social-economic status are particularly important factors that influence SARS-CoV-2 susceptibility and severity [2,3,4,5]. 

At the beginning of the pandemic, the main goal of health authorities was to estimate both morbidity and mortality to predict the trend of the epidemic and provide appropriate healthcare for the population. However, initially, patients with mild and/or moderate symptoms were not tested for SARS-CoV-2 due to a lack of health resources: most of these cases were not included in the epidemiological data [1]. 

The incidence of HIV infection in Brescia is estimated to be one of the highest in Italy, (5.8/100,000 before the pandemic, compared to 4.7/100,000 in the whole country) [6]. Furthermore, patients with HIV (PLWH) were followed up in a single healthcare center: the Spedali Civili di Brescia General Hospital [7]. Especially during the early phases of the pandemic, an appropriate emergency response was mandatory to maintain the delivery of HIV care and protect our PLWH from COVID-19 [8].

Discordant results are available about whether PLWH have an increased risk for COVID-19 and its more severe complications because of their immunodeficiency. Moreover, the higher prevalence of comorbidities in aging PLWH, compared to the general population (such as cardiovascular diseases, chronic respiratory diseases, diabetes, obesity, and cancer) increases vulnerability to COVID-19 and could be associated with more severe COVID-19 symptoms, complications, and fatalities in this population [9,10,11].

Ethnic minorities and migrants are also considered vulnerable population groups since they frequently live in overcrowded facilities such as shelters for the homeless, prisons, and densely populated neighborhoods. Furthermore, migrants and minorities frequently have a generally lower socioeconomic level [12,13]. Due to the above-mentioned conditions, recommendations to prevent the transmission and spread of COVID-19, including social isolation and quarantine, are frequently difficult, if not impossible, to maintain. Moreover, there seem to be inequalities regarding the impact of the COVID-19 pandemic on migrants compared to the general population in the European WHO region [14]. Ultimately, due to their living conditions as well as the cultural and linguistic obstacles they have to overcome to access information and health services, migrants represent a population particularly at risk for contracting COVID-19 and not receiving adequate treatment or vaccination [15]. Notably, Brescia is one of the Italian cities with the highest number of migrants, of whom a significant amount are PLWH [16]. 

Since early 2021, COVID-19 vaccines have been available, and the European Center for Disease Control (ECDC) recommended a multi-tiered approach to COVID-19 vaccine distribution, prioritizing groups considered at high risk for evolution to a more severe form of COVID-19 [17]. In Italy, the COVID vaccination campaign started on 27 December 2020. Initially, the target population consisted of healthcare and social care workers, residents in long-term care facilities, subjects over 80 years and people affected by chronic diseases between the ages of 60 and 79. It was then gradually offered to all lower age groups.

On 10 March 2021, the Italian government issued recommendations for the use of the BNT162b2 vaccine for the vaccination of PLWH regardless of their age [18,19]. Our center immediately responded and organized the COVID-19 vaccination campaign for PLWH in our province through the dedicated vaccination facility that continuously offers all recommended vaccines for PLWH inside our outpatient clinic. The earliest vaccination sessions prioritized the most fragile patients and particularly those with AIDS or a T-cell CD4+ level inferior to 250 cells/mL. Data, however, are lacking about the effectiveness of providing continuous clinical care for these subjects, the risk of COVID-19 and COVID-19 vaccination coverage.

This population-based, retrospective study aimed to assess COVID-19 incidence and vaccination coverage among PLWH and migrants in a local healthcare area in Northern Italy in comparison to the general population during the pandemic.

## 2. Methods

### 2.1. Setting

This study was conducted in the Brescia Province, located in the Lombardy Region (Northern Italy). The province has an area of 3465 km² and a population of 1,255,709 inhabitants. 

The Italian National Health System provides universal coverage, and it is structured on three organizational levels: the central (the Ministry of Health), the regional and the local level. Locally, the Health Protection Agency (HPA) provides both outpatient and inpatient care. The province of Brescia has two HPA: “Brescia” HPA (which includes 90% of inhabitants) and “Montagna” HPA. Brescia Health Protection Agency database, formerly known as the Brescia Local Health Agency database (BLHADB), is a comprehensive and integrated information system, including several databases that track information for every individual that has access to medical services in the Brescia HPA area [8].

We conducted a retrospective cohort study involving all citizens (aged >18 years old) residing in the province of Brescia and connected to the Brescia HPA. We collected the following data from the BLHADB: age, gender, country of origin, comorbidities, and HIV infection. We considered foreigners as all inhabitants with non-Italian citizenship at enrollment. We identified ten groups of chronic diseases using a set of ICD9-CM codes, and an individual was considered to have a chronic disease if he/she had Access to Continuity of Care (ACC) for one or more chronic diseases, as previously described [8].

To describe the SARS-CoV-2 burden, we included all inhabitants part of the Brescia HPA on 15 February 2020. To assess COVID-19 prevalence, we considered infected all persons with a confirmed positive nasopharyngeal swab for SARS-CoV-2 by real-time polymerase chain reaction (RT-PCR) with a European Emergency Use Authorization (EUA).

To describe vaccination coverage, we included inhabitants recorded in the Brescia HPA on 1 January 2021 (thus excluding dead patients and foreigners that definitively returned to their original countries). Fully vaccinated is defined as having received three doses of the vaccines that were used in Italy for the vaccination campaign (BNT162b2, mRNA-1273 and ChAdOx1). ChAdOx1 were used only for the first and the second dose but was eventually dismissed before the third dose. For subjects who received the Ad26.COV2.S vaccine as their first dose, the second dose was considered as the booster dose, and thus, this group was considered fully vaccinated after the administration of the second dose.

### 2.2. Statistical Analysis 

The non-parametric Mann–Whitney test and the chi-square test were used to investigate the statistical significance of the differences between numerical and categorical variables, respectively. The odds ratios (ORs) for SARS-CoV-2 positivity and the ORs, among subjects with confirmed COVID-19, for admission to a hospital department, admission to ICU and death were calculated using multiple logistic regression. HIV status, sex, age category (18–49, 50–69 and >70), citizenship and number of chronic diseases (0, 1, 2–3 and >3) were included in the logistic model. The same model was used to compare the ORs for the propensity to receive at least one dose of the vaccine among Italians and foreigners. The confidence intervals were computed at the 95% level. All statistical tests were two-sided, assumed a level of significance of 0.05 and were performed using Stata 17 software (StataCorp. 2021. Stata Statistical Software: Release 17. College Station, TX, USA: StataCorp LLC.).

This study involving human subjects was performed in accordance with the Helsinki Declaration of 1975 as revised in 2013, and it was approved by the local ethical committee of the Spedali Civili General Hospital of Brescia (approval code NP 5297, local ethical committee approval date 15 March 2022).

## 3. Results

### 3.1. Study Population and SARS-CoV-2 Prevalence

**Figure 1:**
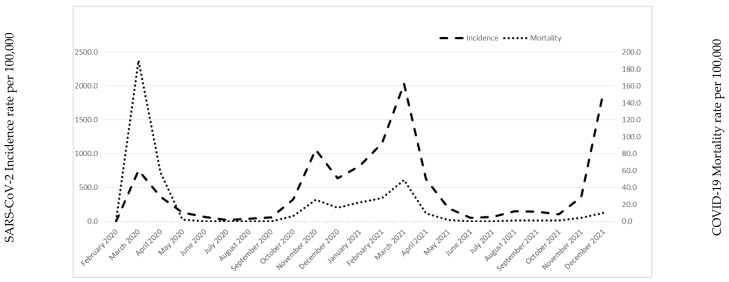
Incidence of SARS-CoV-2 and COVID-19 mortality rates.

A total of 1,004,210 persons aged >18 years residing in the Brescia HPA area were included in the study. Of these, 3817 (0.38%) were PLWH and 134,492 were foreigners (13.39%). As of 31 December 2021, we identified 111,319 (11%) inhabitants with a confirmed positive swab for SARS-CoV-2 and 2936 reinfections (2.9%). The monthly trend of incidence and mortality per 100,000 inhabitants between February 2020 and December 2021 is shown in Figure 1. It is important to underline four peaks or waves of incidence and mortality rates that occurred during the COVID-19 pandemic: March–April–May 2020, October–November–December 2020, March–April–May 2021 and November–December 2021. It is possible to observe that, at the onset of the first wave of the COVID-19 pandemic, there was a higher mortality peak (March 2020) with a relatively lower incidence. On the other hand, the lowest values of incidence and mortality were observed in July–August 2020 (the period between the first and second wave).

Table 1 describes the demographic characteristics of foreigners and PLWH compared with the general population in the Brescia HPA area. Supplementary Table S1 describes the demographics of Italians and foreigners according to HIV-infection status. Almost half of the participants were males (49%), and the median age was 53.7.

In total, 20.1% of Italian citizens showed one comorbidity and 18.8% two or three comorbidities, while 76.9% of foreigners had no history of chronic diseases. Among PLWH, 24.4% had two or three comorbidities and 7.2% more than three relevant diseases. 

In total, 10.89% (12,133/111,319) of patients with SARS-CoV-2 infection were foreigners and 0.3% (346/111,319) PLWH. Forty-three foreigners had HIV infection. During the study period, SARS-CoV-2 infection (11.4% vs. 9% *p* < 0.001), hospitalizations (1.6% vs. 0.9% *p* < 0.001), UCI admission (1.5% vs. 1.1% *p* = 0.002) and death (4.3% vs. 0.5% *p* < 0.001) were statistically more frequent in Italians than in foreigners. SARS-CoV-2 infection (11.1% vs. 9.1%, *p* < 0.001) and deaths (3.9% vs. 1.7%, *p* = 0.04) were more frequent in HIV-uninfected people than in PLWH. 

### 3.2. Burden of HIV-Positive Patients and Migrants with COVID-19

When controlling for age, sex, number of comorbidity and citizenship, PLWH were less likely to have a SARS-CoV-2 diagnosis compared to HIV-negative patients (OR: 0.77; 95% CI 0.69–0.80, *p* < 0.001) while foreigners were less likely to have a SARS-CoV-2 diagnosis compared to native population (OR: 0.71; 95% CI 0.70–0.73, *p* < 0.001) (Table 2).

PLWH also had a similar likelihood of hospitalization (OR: 1.15; 95% CI 0.85–1.56), admission to the ICU (OR: 0.72; 95% CI 0.30–1.76) or death (OR: 0.61; 95% CI 0.26–1.41) compared to HIV-negative patients (Table 3, Table 4 and Table 5).

In contrast, migrants were more likely to be hospitalized (OR: 1.97; 95% CI 1.84–2.12, *p* < 0.001) or admitted to the ICU (OR: 1.83; 95% CI 1.50–2.19) but with a lower risk of death (OR: 0.60; 95% CI 0.46–0.60, *p* < 0.001) compared to Italians (Table 3, Table 4 and Table 5). A sensitivity analysis performed after eliminating subjects aged >60 years old (n. 343,763 subjects) did not show any significant difference from our previous results (data have not been included in this paper).

### 3.3. Vaccination Coverage

On 31 December 2021, we observed a 2.5% decrease in inhabitants referred to Brescia HPA compared to 15 February 2020 (13,531 inhabitants dead and 11,988 moved their residence out of Brescia HPA during this period). The remaining 978,691 subjects were included in the study to assess vaccination coverage. The COVID-19 vaccination trend was similar between PLWH and people without HIV infection; on the contrary, the COVID-19 vaccination trend was slower in foreigners than in Italian citizens.

**Figure 2:**
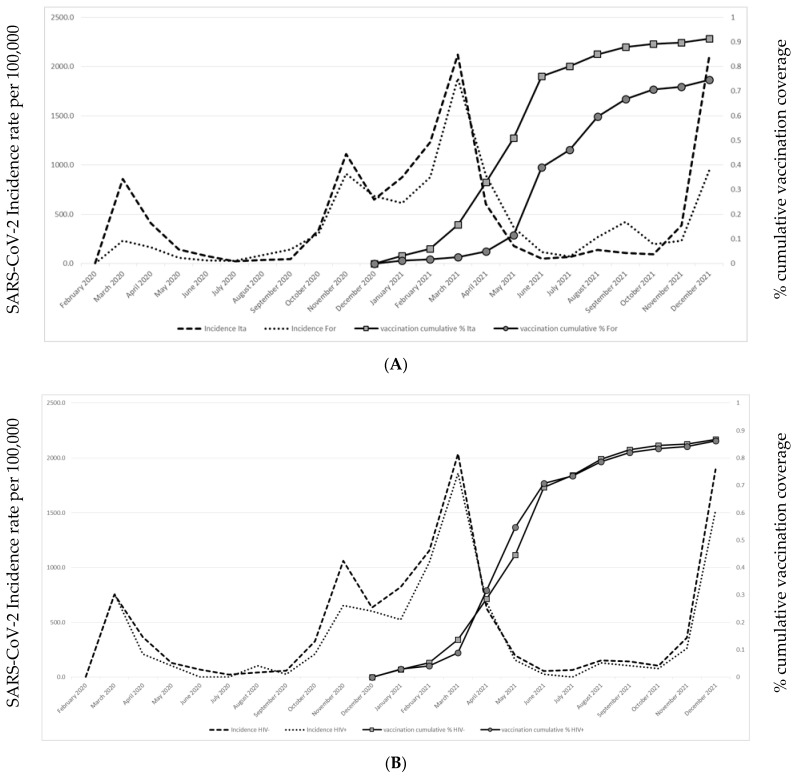
Incidence rate of SARS-CoV-2 infection and cumulative COVID-19 vaccination coverage among Italian citizens (**A**) and among PLWH (**B**).

Vaccination coverage (at least one vaccine administration) and cumulative monthly SARS-CoV-2 infection incidence trend in foreigners and PLWH compared to the general population is shown in Figure 2A,B.

Vaccine shots administered according to citizenship and HIV infection are shown in Table 6. On 31 December 2021 (the last date for which vaccinations were considered in this study), the cumulative numbers of vaccine shots in Brescia were 872,100, 843,825 and 646,253 for the first, second and third dose, respectively. In total, 89.1% of inhabitants had received at least one dose of vaccine, while 70.4% (599,891/851,187) of Italian citizens and 36.3% (46,367/127,504) of foreigner subjects had received three doses of vaccine (*p* < 0.001). Moreover, 8.7% (74,219/851,187) of the Italian subjects and 25.3% (32,372/127,504) of foreigners had not received any dose of vaccine (*p* < 0.001). Only 11% (408/3703) of PLWH had not received any dose of vaccine.

Only a few differences, although statistically significant, were found between PLWH and HIV-uninfected citizens regarding vaccination coverage both in Italians and foreigners. Among Italian PLWH, only 9.3% were unvaccinated and 72.4% had received three doses, confirming that vaccination coverage was very similar to that of Italians without HIV infection (8.7% vs. 70.5%, respectively). Among foreigners, COVID-19 vaccine coverage was lower: foreign PLWH not vaccinated at the time of the study was 21.5%, and only 49.4% had received three doses (Table 6).

Finally, we employed a multivariate logistic model to identify characteristics associated with COVID-19 vaccine compliance (at least 1 dose on 31 December 2021) among foreigners and Italian citizens controlled by age, sex and number of comorbidities (Table 7). Among Italians, we found that males (vs. females; OR: 0.97; 95% CI 0.96–0.99, *p* < 0.001), people aged 50–69 (vs. <50; OR: 0.93; 95% CI 0.91–0.94, *p* < 0.001), aged >70 (vs. <50; OR: 0.94; 95% CI 0.92–0.96, *p* < 0.001) and people with comorbidities were more likely to be vaccinated. However, PLWH were less likely to be vaccinated (OR: 1.17; 95% CI 1.04–1.32, *p* < 0.01).

Among foreigners, male gender (vs. female, OR: 0.85; 95% CI 0.83–0.88, *p* < 0.001), and the number of comorbidities increased the probability of undergoing COVID-19 vaccination. Of note, vaccination coverage decreased with age (foreigners aged 50–69 years vs. <50, OR: 1.30; 95% CI 1.26–1.34, *p* < 0.001, and foreigners aged >70 years vs. <50, OR: 3.51; 95% CI 3.34–3.37, *p* < 0.001). Finally, foreigners with HIV infection were more likely vaccinated compared to those without HIV infection (OR: 0.87; 95% CI 0.7–1.98–3.37), although the difference did not reach statistical significance.

## 4. Discussion

This is a population-based, registry-linked retrospective study of vulnerable populations, such as foreigners and PLWH, residing in the HPA of Brescia and followed between January 2020 and December 2021 during the COVID-19 pandemic to evaluate the risk of SARS-CoV-2 infection, hospitalization, and death and assess COVID-19 vaccination coverage. 

In our study, foreigners showed a lower risk than the general population of having a SARS-CoV-2 diagnosis or dying from COVID-19. Nonetheless, COVID-19 vaccine hesitancy was higher among foreigners than Italians. In contrast, the risk of infection, hospitalization and death, and vaccine coverage among PLWH were similar to that of the general population in our area. Moreover, the likelihood of not being vaccinated among Italians was significantly higher in citizens aged <50 years, females, PLWH, and people without chronic comorbidities. Among foreigners, the risk was higher among females and in people without chronic diseases, but in contrast to Italians, the risk of not being vaccinated was higher in people more than 50 years old (higher risk still for those aged >70). PLWH, both Italians and foreigners, were less hesitant toward COVID-19 vaccinations than the HIV-uninfected population.

Considering all residents referred to the HPA of Brescia, we observed a lower number of confirmed SARS-CoV-2 infections in people older than 50 years compared to younger categories. This was probably because younger subjects are usually workers and, as such, have greater possibilities and obligations to be tested for SARS-CoV-2 and to get vaccinated. Furthermore, people with at least one comorbidity (and thus considered at high risk for severe COVID from the beginning of the pandemic) had an increased risk for SARS-CoV-2 diagnosis. The number of infections reported during the first wave was lower than in later ones, but the number of deaths reported during the first wave was significantly higher. 

There is a certain level of uncertainty about the real number of infections reported during the first wave, as only severe cases were appropriately tested, and contact tracing was suboptimal between March and April 2020 [20]. Initially, testing for SARS-CoV-2 was mostly limited to serve as a confirmation for clinical COVID-19. Tests were extensively used as a tracking strategy only after the first lockdown [21].

Regarding foreigners, the OR for SARS-CoV-2 infection was significantly lower, but the ORs for hospitalization and ICU admission were higher than among Italian citizens. Notwithstanding, the risk of death was higher in foreigners than in Italian citizens.

SARS-CoV-2 infection diagnosis could be less achievable for foreigners due to misinformation, language barriers, and lack of trust in traditional medicine and might explain the fact that only severe cases reached the hospitals (mainly during the first wave). Pagani et al. performed a cross-sectional study in a social-housing neighborhood in Milan where 30% of inhabitants are non-Italian, and they found that the prevalence of anti-SARS-CoV-2 nucleocapsid antibodies in foreigners was more than two-fold higher than among Italians (23% vs. 9%) [4]. Foreigners seem to be more reluctant to vaccination, especially women and people > 50 years of age [22]. One possible explanation for this is that they are a minority that is frequently jobless and, therefore, can easily avoid SARS-CoV-2 testing and vaccination.

In our study, HIV infection was not associated with a higher risk of SARS-CoV-2 infection, hospital admission, severe disease or death compared to the general population in the adjusted analysis. A recent meta-analysis showed that PLWH have an increased risk of hospital admission for COVID-19 (OR: 1.49; 95% CI 1.01–2.21) and risk of mortality (OR: 1.76, 95% CI 1.31–2.35; adjusted for age and sex), but HIV infection does not increase the likelihood of having severe COVID-19 (OR: 1.28; 95% CI 0.77–2.13) [23]. Our results also suggest an increased risk for hospital admission due to COVID-19 in HIV-infected individuals although without reaching statistical significance. This possibly reflects the conservative approach applied to PLWH (given the inconsistent evidence regarding their outcome and their known vulnerability to infections due to immunodeficiency) to prevent any potential complications. Notwithstanding, our data show that the risk of severe COVID-19 or death was not increased in PLWH compared to the general population. Moreover, COVID-19 vaccine hesitancy was not different between PLWH (11%) and HIV-uninfected people (10.89%). Our center promptly added the COVID-19 vaccination service to the HIV-related health service, and PLWH were actively prompted to receive vaccination since April 2021. Interestingly, even though foreign PLWH were more hesitant toward vaccination than Italian PLWH, they were less hesitant than HIV-uninfected foreigners, showing that, in our area, these patients closely follow and trust healthcare professionals. A recent study performed in the State of New York found that COVID-19 vaccine coverage among PLWH was lower than among the general population (63.5% vs. 75%), and this disparity remained after examining ethnics subgroups [24,25].

Our study has both strengths and limits. This study was conducted on a single-province cohort, but it is noteworthy that this HPA has a total population of more than 1.2 million inhabitants as well as one of the highest incidences of HIV infection and a significant proportion of migrants among the provinces of northern Italy. Furthermore, Brescia was one of the provinces hit the hardest by COVID-19 in the whole European area. BHPADB is a large, population-based comprehensive and integrated information system that has the usual limits of currently available large databases of health data coming from various sources. Nevertheless, a validation study and various analyses have shown a reasonably high quality of the database. This population-based approach allows estimating incidence, prevalence, mortality rates and COVID-19 vaccine coverage for all residents living in the area, avoiding selection bias.

Regarding the limits of this study, we have not separately studied ethnic minorities, country of origin or analyzed individual-level socioeconomic information as possible factors influencing SARS-CoV-2 infection or vaccine hesitancy. Moreover, clinical aspects such as CD4+ cell count or chronic therapies were not evaluated.

Conclusively, in our study, we have observed fewer SARS-CoV-2 infections among migrants, possibly because they had less access to SARS-CoV-2 diagnostics. Nonetheless, they were at higher risk of hospitalization and ICU admission but not of dying. Overall, significant statistical differences were not observed comparing PLWH and the general population. Regarding COVID-19 vaccination coverage, foreigners were more hesitant. 

These findings clearly suggest the need for further studies to identify key specific social determinants of health that contribute to the high risk of confirmed SARS-CoV-2 infection in some groups to facilitate prevention and treatment efforts.

## Figures and Tables

**Table 1:** Descriptive characteristics of the study population by citizenship and HIV status.

	Total	CITIZENSHIP		*p*	HIV STATUS		*p*
		Italian	Foreigner		Positive	Negative	
**N (%)**	1,004,210	869,718 (86.6%)	134,492 (13.4%)		3817 (0.38%)	1,000,393 (99.62%)	
**Age, median (p25-p75)**	51.6 (37.6–66.3)	53.7 (39.6–68.5)	40.6 (31.5–50.8)	<0.001	54 (46.5–58.9)	51.6 (37.6–66.3)	0.02
**Age category**							
**18–49 N (%)**	469,586 (46.8%)	370,599 (42.6%)	98,987 (73.6%)	<0.001	1311 (34.3%)	468,275 (46.8%)	<0.001
**50–69 N (%)**	266,510 (26.5%)	239.088 (27.5%)	27,422 (20.4%)		2140 (56.1%)	264,370 (26.4%)	
**>70 N (%)**	268,114 (26.7%)	260,031 (29.9%)	8083 (6.0%)		366 (9.6%)	267,748 (26.8%)	
**Male, N (%)**	492,625 (49.1%)	427,237 (49.1%)	65,388 (48.6%)	0.001	2717 (71.2 %)	489,908 (49.0%)	<0.001
**Comorbidity**							
**none**	563,589 (56.1%)	461,536 (53.1%)	102,053 (76.9%)	<0.001	1679(44.0%)	561,910 (56.2%)	<0.001
**1**	196,298 (19.5%)	175,370 (20.1%)	20,928 (15.6%)		933 (24.4%)	195,365 (19.5%)	
**2–3**	173,346 (17.3%)	163,761 (18.8%)	9585 (7.1%)		932 (24.4%)	172,414 (17.2%)	
**>3**	70,977 (7.1%)	69,051 (8.0%)	1926 (1.4%)		273 (7.2%)	70,704 (7.1%)	
**Citizenship**							
**Italian**		-	-		3284 (86.0%)	866,434 (86.6%)	n.s.
**non-Italian**		-	-		533 (14.0%)	133,959 (13.4%)	
**HIV status**					-	-	
**Positive**		3284 (0.4%)	533 (0.4%)	n.s.	-	-	
**Negative**		866,434 (99.6%)	133,959 (99.6%)				
**COVID-19 positive cases**	111,319 (11.1%)	99,186 (11.4%)	12,133 (9.0%)	<0.001	346 (9.1%)	110,973 (11.1%)	<0.001
**Admitted to hospital**	15,588 (1.6%)	14,374 (1.6%)	1214 (0.9%)	<0.001	59 (1.6%)	15,529 (1.6%)	n.s.
**Admitted to ICU**	1594 (0.2%)	1459 (1.5%)	135 (1.1%)	0.002	5 (1.5%)	1591 (1.4%)	n.s.
**Death**	4297 (0.4%)	4239 (4.3%)	58 (0.5%)	<0.001	6 (1.7%)	4292 (3.9%)	0.04
**N (%) of reinfections**	2936 (0.3%)	2632 (2.7%)	304 (2.5%)	n.s.	5 (0.9%)	2932 (2.7%)	0.04

n.s. = not significant.

**Table 2:** Multivariate logistic model of risk factors associated with COVID-19 diagnosis.

	COVID-19 DIAGNOSIS		OR	95% CI	*p*
	Yes	No			
**Age category**					
18–49 N	56,982 (51.2%)	412,604 (46.2%)	1 reference		
50–69 N	28,731 (25.8%)	237,779 (26.6%)	0.80	0.79–0.82	<0.001
>70 N	25,606 (23.0%)	242,508 (27.2%)	0.60	0.59–0.61	<0.001
**Gender**					
Female	56,183 (50.5%)	455,402 (51.0%)	1 reference		
Male	55,136 (49.5%)	437,489 (49.0%)	1.01	0.99–1.02	n.s
**Comorbidity**					
none	62,339 (56.0%)	501,250 (56.1%)	1 reference		
1	21,370 (19.2%)	174,928 (19.6%)	1.09	1.07–1.11	<0.001
2–3	18,286 (16.4%)	155,060 (17.4%)	1.20	1.17–1.22	<0.001
>3	9324 (8.4%)	61,653 (6.9%)	1.67	1.63–1.72	<0.001
**Citizenship**					
Italian	99,186 (89.1%)	770,532 (86.3%)	1 reference		
Foreigner	12,133 (10.9%)	122,359 (13.7%)	0.71	0.70–0.73	<0.001
**HIV status**					
negative	110,973 (99.7%)	889,420 (99.6%)	1 reference		
positive	346 (0.3%)	3471 (0.4%)	0.77	0.69–0.86	<0.001

**Table 3:** Multivariate logistic model of risk factors associated with hospitalization in patients with COVID-19.

	COVID-19 Cases Admitted to Hospital (Any Department)		OR	95% CI	*p*
	Yes	No			
**Age category**					
18–49	1736 (11.1%)	55,246 (57.7%)	1 reference		
50–69	3644 (23.4%)	25,087 (26.2%)	3.63	3.41–3.86	<0.001
>70 N	10,208 (65.5%)	15,398 (16.1%)	11.30	10.58–12.07	<0.001
**Sex**					
Female	6456 (41.4%)	49,727 (51.9%)	1 reference		
Male	9132 (58.6%)	46,004 (48.1%)	1.81	1.74–1.88	<0.001
**Comorbidity**					
none	2916 (18.7%)	59,423 (62.1%)	1 reference		
1	2863 (18.4%)	18,507 (19.3%)	1.83	1.73–1.94	<0.001
2–3	5489 (35.2%)	12,797 (13.4%)	2.94	2.78–3.12	<0.001
>3	4320 (27.7%)	5004 (5.2%)	4.29	4.02–4.58	<0.001
**Citizenship**					
Italian	14,374 (92.2%)	84,812 (88.6%)	1 reference		
Foreigner	1214 (7.8%)	10,919 (11.4%)	1.97	1.84–2.12	<0.001
**HIV status**					
negative	15,529 (99.6%)	95,444 (99.7%)	1 reference		
positive	59 (0.4%)	287 (0.3%)	1.15	0.85–1.56	n.s

n.s.= not significant.

**Table 4:** Multivariate logistic model of risk factors associated with ICU admission in patients with COVID-19.

	COVID-19 Cases Admitted to ICU		OR	95% CI	*p*
	Yes	No			
**Age category**					
18–49 N (%)	157 (9.8%)	56,825 (51.8%)	1 reference		
50–69 N (%)	485 (30.4%)	28,246 (25.7%)	4.68	3.88–5.65	<0.001
>70 N (%)	954 (59.8%)	24,652 (22.5%)	8.43	6.91–10.27	<0.001
**Sex**					
Female	439 (27.5%)	55,744 (50.8%)	1 reference		
Male	1157 (72.5%)	53,979 (49.2%)	2.86	2.56–3.2	<0.001
**Comorbidity**					
none	277 (17.4%)	62,062 (56.6%)	1 reference		
1	382 (23.9%)	20,988 (19.1%)	2.48	2.11–2.92	<0.001
2–3	615 (38.5%)	17,671 (161%)	3.03	2.58–3.57	<0.001
>3	322 (20.2%)	9002 (8.2%)	2.53	2.1–3.06	<0.001
**Citizenship**					
Italian	1461 (91.5%)	97,725 (89.1%)	1 reference		
Foreigner	135 (8.5%)	11,998 (10.9%)	1.83	1.50–2.19	<0.001
**HIV status**					
negative	1591 (99.7%)	109,382 (99.7%)	1 reference		
positive	5 (0.3%)	341 (0.3%)	0.72	0.30–1.76	n.s

n.s. = not significant.

**Table 5:** Multivariate logistic model of risk factors associated with death from COVID-19.

	COVID-19 Related Death		OR	95% CI	*p*
	Yes	No			
**Age category**					
18–49 N (%)	26 (0.6%)	56,956 (53.2%)	1 reference		
50–69 N (%)	246 (5.7%)	28,485 (26.6%)	12.61	8.39–18.95	<0.001
>70 N (%)	4026 (93.7%)	21,580 (20.2%)	164.46	110.81–244.09	<0.001
**Sex**					
Female	1713 (39.9%)	54,470 (50.9%)	1 reference		
Male	2585 (60.1%)	52,551 (49.1%)	1.82	1.7–1.94	<0.001
**Comorbidity**					
none	264 (6.1%)	62,075 (58.0%)	1 reference		
1	573 (13.3%)	20,797 (19.4%)	1.96	1.68–2.29	<0.001
2–3	1705 (39.7%)	16,581 (15.5%)	3.12	2.71–3.58	<0.001
>3	1756 (40.9%)	7568 (7.1%)	4.67	4.06–5.38	<0.001
**Citizenship**					
Italian	4239 (98.6%)	94,947 (88.7%)	1 reference		
Foreigner	59 (1.4%)	12,074 (11.3%)	0.60	0.46–0.78	<0.001
**HIV status**					
negative	4292 (99.9%)	106,681 (99.7%)	1 reference		
positive	6 (0.1%)	340 (0.3%)	0.61	0.26–1.41	n.s

n.s. = not significant.

**Table 6:** Vaccinations according to citizenship and HIV infection.

	Total	Foreigners					Total	Italians				
		HIV+		HIV−		*p*		HIV+		HIV−		*p*
		*n*	%	*n*	%			*n*	%	*n*	%	
**Unvaccinated**	32,372 (25.4%)	109	21.60%	32,263	25.40%	<0.001	74,219 (8.7%)	299	9.30%	73,920	8.70%	<0.001
**1 dose**	5160 (4.0%)	12	2.40%	5148	4.10%		19,968 (2.3%)	67	2.10%	19,901	2.30%	
**2 doses**	43,610 (34.2%)	134	26.60%	43,476	34.20%		157,109 (18.5%)	516	16.10%	156,593	18.50%	
**3 doses**	46,362 (36.4%)	249	49.40%	46,113	36.30%		599,891 (70.5%)	2.317	72.40%	597,574	70.50%	
**Total**	127,504	504	100.00%	127,000	100.00%		851,187	3199	100.00%	847,988	100.00%	

**Table 7:** Multivariate logistic model of factors associated with COVID-19 vaccination (at least one COVID-19 vaccine dose).

	Italians					Foreigners				
	At Least One Dose		OR	95% CI	*p*	At Least One Dose		OR	95% CI	*p*
	Yes	No				Yes	No			
**Age category**										
18–49 N (%)	330,598 (42.5%)	36,223 (48.8%)	1 reference			71,871 (75.5%)	21,951 (67.8%)	1 reference		
50–69 N (%)	217,316 (28.0%)	19,834 (26.7%)	0.93	0.91–0.94	<0.001	19,277 (20.3%)	6935 (21.4%)	1.30	1.26–1.34	<0.001
>70 N (%)	229,054 (29.5%)	18,162 (24.5%)	0.94	0.92–0.96	<0.001	3984 (4.2%)	3486 (10.8%)	3.51	3.34–3.7	<0.001
**Sex**										
Female	395,501 (50.9%)	37,856 (51.0%)	1 reference			47,969 (50.4%)	17,718 (54.7%)	1 reference		
Male	381,467 (49.1%)	36,363 (49.0%)	0.97	0.96–0.99	<0.001	47,163 (49.6%)	14,654 (45.3%)	0.85	0.83–0.88	<0.001
**Comorbidity**										
none	410,126 (52.8%)	46,975 (63.3%)	1 reference			71,264 (74.9%)	25,317 (78.2%)	1 reference		
1	160,425 (20.6%)	12,665 (17.1%)	0.70	0.69–0.72	<0.001	15,526 (16.3%)	4441 (13.7%)	0.70	0.67–0.72	<0.001
2–3	148,162 (19.1%)	9877 (13.3%)	0.60	0.59–0.62	<0.001	6943 (7.3%)	2193 (6.8%)	0.61	0.58–0.65	<0.001
>3	58,255 (7.5%)	4702 (6.3%)	0.73	0.71–0.76	<0.001	1399 (1.5%)	421 (1.3%)	0.47	0.42–0.52	<0.001
**HIV status**										
negative	774,068 (99.6%)	73,920 (99.6%)	1 reference			94,737 (99.6%)	32,263 (99.7%)	1 reference		
positive	2900 (0.4%)	299 (0.4%)	1.17	1.04–1.32	0.01	395 (0.4%)	109 (0.3%)	0.87	0.7–1.08	n.s

n.s. = not significant.

## Data Availability

Not applicable.

## Supplementary Materials

The following supporting information can be downloaded at: https://www.mdpi.com/article/10.3390/life12071009/s1, Table S1: Descriptive characteristics of the study population by HIV status and citizenship.

## Appendix A

Collaborators of HIV-CoV Group: Samuele Storti, Melania Degli Antoni, Giorgio Tiecco, Stefania Arsuffi, Davide Minisci, Francesca Pennati, Marco Di Gregorio, Cosimo Colangelo, Paola Nasta, Emanuele Focà, Ilaria Izzo, Silvia Amadasi, Francesco Castelli, Eugenia Quiros-Roldan. All of the above-mentioned authors have the following affiliation: Department of Infectious and Tropical Diseases, University of Brescia and ASST Spedali Civili General Hospital, Brescia, Italy.
