# Supplementary material for: SARS-CoV-2 Infection and Vaccination Coverage among Fragile Populations in a Local Health Area of Northern Italy
Source: Life (Basel). 2022 Jul 7;12(7):1009. doi: 10.3390/life12071009 (PMC9316873; doi:10.3390/life12071009)
Supplement: Supplementary file 1 [file life-12-01009-s001.zip › life-1688892-supplementary.pdf]

Supplementary Table S1. Descriptive characteristics of the study population by HIV status and citizenship.

	Italians (n=869.718)				Foreigners (n=134.492)			
	TOT	HIV STATUS		p	TOT	HIV STATUS		p
		positive	negative			positive	negative	
N	869.718	3.284	866.434		134.492	533	133.959	
Male, N (%)	427.237 (49,1%)	2.479 (75.5%)	424.758 (49.0%)	< 0,001	65.388 (48.6%)	238 (44.6%)	65.150 (48.6%)	0.07
Age, median (p25-p75)	53,7 (39.6-68.5)	55,1 (48.6-59.6)	53,7 (39.6-68.5)	n.s.	40,6 (31.5-50.8)	44,2 (36.6-51.7)	40.6 (31.5-50.8)	< 0.001
Age category								
18-49 N (%)	370.599 (42.6%)	938 (28.6%)	369.661 (42.6%)		98.987 (73.6%)	373 (70.0%)	98.614 (73.6%)	
50-69 N (%)	239.088 (27.5%)	1.990 (60.6%)	237.098 (27.4%)	< 0,001	27.422 (20.4%)	150 (28,1%)	27.272 (20.4%)	< 0.001
>70 N (%)	260.031 (29.9%)	356 (10.8%)	259.675 (30.0%)		8.083 (6.0%)	10 (1.9%)	8.073 (6.0%)	
Comorbidity								
none	461.536 (53.1%)	1.328 (40.5%)	460.208 (53.1%)		102.053 (75.9%)	351 (65.9%)	101.702 (75.9%)	
1	175.370 (20.2%)	839 (25.5%)	174.531 (20.2%)	< 0,001	20.928 (15.6%)	94 (17.6%)	20.834 (15.6%)	< 0.001
2-3	163.761 (18.8%)	853 (26.0%)	162.908 (18.8%)		9.585 (7.1%)	79 (14.8%)	9.506 (7.1%)	
>3	69.051 (7.9%)	264 (8.0%)	68.787 (7.9%)		1.926 (1.4%)	9 (1.7%)	1.917 (1.4%)	
Covid-19 positive cases	99.168 (11.4%)	303 (9.2%)	98.883 (11.4%)	< 0,001	12.133 (9.0%)	43 (8.1%)	12.090 (9.0%)	n.s.
Admitted to hospital	14.374 (1.7%)	47 (15.5%)	14.327 (14.5%)	n.s.	1.214 (0.9%)	12 (27.9%)	1.202 (9.9%)	< 0.001
Admitted to ICU	1.459 (0.2%)	<3*	1.459 (1.5%)	-	135 (0.1%)	3 (7.0%)	132 (1.1%)	< 0.001
Death	4.239 (0.5%)	5 (1.6%)	4.234 (4.3%)	0,02	58 (0.04%)	<3*	58 (0.5%)	-
N (%) of reinfections	2.632 (0.3%)	4 (1.3%)	2.628 (2.7%)	n.s.	304 (0.2%)	<3*	304 (2.4%)	-

*assuming zero cases when less than three cases reported (<3)
